# Evaluating the Effects of Conventional Polyethylene and Biodegradable Film Mulching on Soil Fauna Abundance and Diversity in a Rice Cropping System

**DOI:** 10.3390/toxics14080651

**Published:** 2026-07-24

**Authors:** Tianmu Peng, Sumaira Gul, Fengshuo Huang, Yang Zhao, Licheng Peng, Muhammad Amjad Khan, Rafia Zainab, Peng Guo, Tauseef Ahmad, Junhua Yin, Zongyin Zhang, Qin Liu, Mingfu Lyu, Yin Xie, Qing Huang

**Affiliations:** 1 Key Laboratory of Agro-Forestry Environmental Processes and Ecological Regulation of Hainan Province/School of Environmental Science & Engineering/School of Tropical Agricultural and Forestry/Hainan Province Flexible Talent Introduction Collaborative Innovation Center/International Joint Research Center for the Control and Prevention of Environmental Pollution on Tropical Islands of Hainan Province, Hainan University, Haikou 570228, China; ptm0905@163.com (T.P.); gsumaira831@gmail.com (S.G.); fengshuo20210305@163.com (F.H.); zhaoyang1376@163.com (Y.Z.); lcpeng@hainanu.edu.cn (L.P.); amjadkhan_best@yahoo.com (M.A.K.); rafiaz160@gmail.com (R.Z.); tauseef.hainu@gmail.com (T.A.); 2 SINOPEC (Beijing) Research Institute of Chemical Industry Co., Ltd., Beijing 100013, China; guopeng.bjhy@sinopec.com (P.G.); zhangzy.bjhy@sinopec.com (Z.Z.); lumf.bjhy@sinopec.com (M.L.); 3 Shandong Qingtian Plastic Co., Ltd., Zibo 255410, China; yjh13668630343@163.com; 4 Institute of Environment and Sustainable Development in Agriculture, Chinese Academy of Agricultural Sciences, Beijing 100081, China; liuqin02@caas.cn; 5 Agricultural Ecology and Resource Protection Station of Hainan Provincial, Haikou 570100, China; 18509368556@163.com

**Keywords:** film mulching, cropping system, ecological impacts, soil fauna, sustainability

## Abstract

Biodegradable film mulching has been developed as promising alternative to overcome the adverse effects posed by conventional polyethylene film mulching in cropping systems. However, evidence regarding whether conventional polyethylene and biodegradable film mulching affect soil fauna in cropping systems still remains unknown. This study was therefore designed to examine the differences in the responses of soil fauna and selected physicochemical parameters under different mulch treatments, i.e., polyethylene film mulch (PE), biodegradable film mulches (BMs1–5), biodegradable paper (BP) and no-mulch control (CK), in a rice cropland. The results demonstrated that all mulch treatments significantly increased soil temperature (*p* < 0.001) and reduced soil pH (*p* < 0.05) and electrical conductivity (*p* < 0.01) without significantly affecting soil organic content and water content compared to CK. Regarding soil fauna, during the growing stage, the abundance of soil fauna under all of the mulch treatments declined (39%) compared to CK. However, during the harvesting stage, soil faunal abundance was 54% and 45% higher under BP and BMs, respectively, compared with PE. No significant differences were observed in richness, diversity, or evenness among the mulch treatments. This study provides insight into the benefits of environmentally friendly film mulching in terms of biodiversity conservation and soil health.

## 1. Introduction

Mulching has been employed in agriculture since the 1920s, when paper sheets were first introduced as a soil-covering material, and since then, it has become a widely adopted agricultural practice worldwide [1]. In recent decades, the use of mulching has expanded significantly in modern farming because of its effectiveness in improving crop yield and the quality of horticultural production, particularly in cold and arid regions [2]. By reducing soil water evaporation and moderating soil temperature, mulching enhances soil moisture conservation and thermal insulation, creating favorable conditions for plant growth, especially under low-temperature or water-limited environments [3]. Improved soil temperature and moisture also stimulate microbial abundance and activity, which enhances nutrient cycling and increases nutrient availability for plant uptake, thereby supporting crop productivity [4]. Furthermore, mulching suppresses weed emergence and growth, reducing competition with crops for water, nutrients, and other essential resources [5].

Despite its benefits for crop production, residual polyethylene (PE) mulch film has led to growing environmental concerns. PE is the most commonly used mulch in commercial cropping systems [6]. PE is prevalent due to its easy availability, low cost, flexibility, durability, and ease of mechanical application on an industrial level [7]. However, the extensive application of conventional PE has led to the accumulation of residual films in the soil environment [7,8], because it is resistant to degradation in the natural environment, which in turn further affects crop growth, microorganisms, and earthworms in the soil [9,10,11]. The lingering presence of PE in the fields over time can be ingested or carried by soil fauna, affecting their abundance and diversity, reproduction, growth rates, immunity, and nutrition [12,13,14].

Biodegradable film mulches (BDMs) have been developed as promising alternatives to lessen adverse effects posed by conventional mulches in cropland [15]. BDMs are typically produced from natural feedstocks, including plant fibers and starch, as well as biodegradable and biocompatible polymers such as poly(butylene adipate-co-terephthalate) (PBAT), polylactic acid (PLA), and polyglycolic acid (PGA) [16]. In contrast to PE mulch, they can naturally degrade after use and become integrated into the soil with the help of soil organisms into microbial biomass, water and carbon dioxide [17,18]. They also lessen some adverse effects posed by conventional PE, such as reduced soil organic matter content, extreme use of soil nutrients and heat stress [19]. However, the degradation of BDMs under field conditions is highly variable and depends on multiple factors, including polymer composition, film thickness, soil properties, microbial activity, and the timing of incorporation [15]. Research by Blaise et al. has demonstrated that BDMs have significant positive effects on soil biological activity, microbial diversity, and abundance [20]. Therefore, advancing sustainable agricultural practices requires strategies that promote soil biodiversity and leveraging their associated ecological services.

As one of the key ecological regulators of agroecosystems, soil fauna plays an essential role in providing ecosystem services such as soil organic matter decomposition, carbon sequestration, nutrient cycling, improving biological soil fertility, enhancing crop productivity and maintaining soil quality [21,22]. Among soil fauna, macrofauna constitutes the predominant portion of total soil animal biomass and plays a significant role in sustaining soil food-web functionality, as documented by [23]. Soulé et al. have documented a continuous and alarming decline in biodiversity within cropped agroecosystems, encompassing reductions in both species’ abundance and diversity [24]. Research conducted by Culman et al. has revealed that intensified agricultural methods result in the loss of both aboveground and belowground biodiversity [25]. Although film mulching has been widely studied for its effects on soil temperature, moisture conservation, crop growth, and microbial activity, it remains unclear whether mulch-induced hydrothermal changes drive taxon-specific responses of soil fauna. Soil fauna are ectothermic, and many groups are sensitive to soil microclimatic changes, particularly temperature and moisture; therefore, their abundance and activity can respond strongly to variations in soil temperature and water availability [26,27,28]. Therefore, this study aimed to assess the effects of different mulch treatments, i.e., polyethylene mulch (PE), biodegradable mulches (BMs1-5) and paper mulch (BP), on the diversity and abundance of soil fauna in a rice cropland. We hypothesized that (1) mulch-induced warming during the growing stage would reduce the abundance of moisture-sensitive soil fauna compared with CK and (2) biodegradable films and paper would show lower disturbance than PE at harvest because degradation weakens their warming effect. This study will provide a more realistic and comprehensive understanding of environmentally friendly film mulching and insights into the benefits of sustainable farming practices that contribute to conserving soil biodiversity and maintain ecosystem functionality.

## 2. Materials and Methods

### 2.1. Site Description

**Figure 1:**
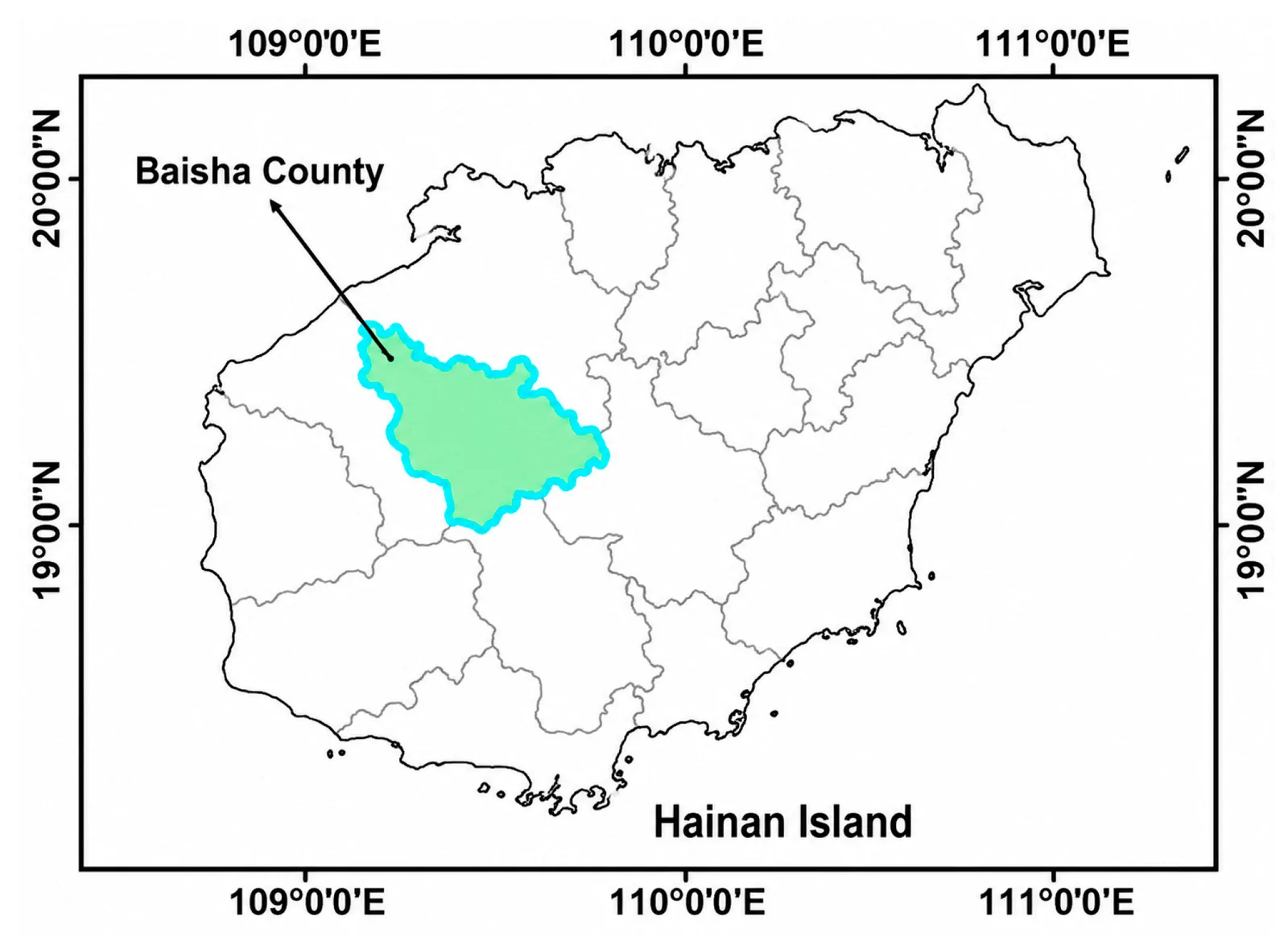
Study map of Hainan Island and Baisha County. The colored view of Hainan Island shows the location of Baisha County, reproduced from [31].

The selected study site is located at Qingsong township of Baisha County, Hainan Province, South China [19°13′27″ N, 109°26′50″ E] (https://geohack.toolforge.org/geohack.php?params=19.224172222222_N_109.44709722222_E_globe:earth&language=en, accessed on 16 July 2026) as shown in Figure 1. The region is characterized by a tropical monsoon climate and has hot and humid summers and dry, mild winters. Throughout the year, an average temperature typically fluctuates between 17 °C and 36 °C. The region receives an annual precipitation ranging between 1500 and 2000 mm, with 161.37 rainy days (44.21% of the time) annually according to Wang and Meng [29].

The soil at this site was classified as a Hydragric Anthrosol according to the World Reference Base (WRB) for Soil Resources [30]. The soil exhibited a medium loam texture, with a neutral pH of 7.18. The average soil bulk density was 1.29 g cm^−3^. The soil organic carbon, soil organic matter, total nitrogen, available nitrogen, and available phosphorous within 0–30 cm topsoil were measured in the laboratory and are presented in Table S1 (see Supplementary Materials). Field temperature and humidity were continuously monitored using a network transmitter (model: RS-4G-2H-V3; Shandong Renke Measurement and Control Technology Co., Ltd., Jinan, China). The collected data is provided in Table S2 (see Supplementary Materials). The study site was planted with rice in January 2023 and harvested in June 2023.

### 2.2. Experimental Design

The experiment consists of eight treatments: (1) a non-biodegradable polyethylene (PE) film mulch, (2) five plots covered with biodegradable mulches (BMs1–5), (3) a biodegradable paper (BP) mulch, and (4) a control (CK) without mulch (Figure S1, see Supplementary Materials). The eight experimental plots with three replications, each with an area of about 100 m^2^ (50 m long and 2 m wide), were organized in a completely randomized block design (Figure S2, see Supplementary Materials). The PE was obtained from SINOPEC (Beijing) Research Institute of Chemical Industry Co., Ltd., China, with thickness of 10 μm. The biodegradable mulches (all 10 μm thickness) were sourced from different commercial manufacturers and differed in polymer composition: BM1 was poly(glycolic acid) (PGA), BM2 was poly(lactic acid) (PLA), BM3 was poly(butylene adipate-co-terephthalate) (PBAT), BM4 was a PGA/PBAT blend, and BM5 was a PLA/PBAT blend. The physical and chemical properties of all film mulches are summarized in Table S3 (see Supplementary Materials).

PLA is widely used in biodegradable film production because of its favorable processability, composability, and mechanical performance [32]. PGA is another biodegradable polyester with high crystallinity, high mechanical strength, and relatively rapid biodegradation, and PGA has been reported to degrade faster than PBAT in blend-film systems [33]. In contrast, PBAT-based biodegradable mulch materials are flexible but may show relatively low tensile strength and a high melt index, which can limit their film-forming performance and practical application in agricultural mulch production [34]. To address these limitations, PBAT is commonly blended with other biodegradable polymers. For instance, PBAT/PLA blends have been shown to significantly enhance tensile strength and reduce melt index compared with neat PBAT, thereby improving film-forming ability and mechanical stability [34]. Similarly, incorporating PGA into PBAT-based systems can promote the degradation rate of the blend while improving the rigidity of PBAT [33]. The biodegradable paper mulch (BP), purchased from a local supplier in Hainan, was composed of Kraft paper with a thickness of 100 μm.

### 2.3. Sample Collection and Physicochemical Analysis

Surface soil (0–30 cm) was collected using a stainless-steel shovel during the growing (March 2023) and harvesting (June 2023) stages. The samples were stored at 4 °C for subsequent measurement of soil physicochemical properties, including soil temperature, pH, electrical conductivity, water content, and soil organic matter, for both the control and treatment plots. pH was determined from a 1:2.5 soil to water ratio using a pH meter (Sartorius PB-10-C, Sartorius AG, Göttingen, Germany). Electrical conductivity was determined from a 1:5 soil to water ratio using an electrical conductivity meter (Mettler Toledo Instrument, model, FG3, Shanghai, China). Soil temperature was recorded at 15 cm soil depths using a soil digital thermometer. Water content was determined gravimetrically through the oven drying method at 105 °C for 24 h [35]. Soil organic carbon (SOC) and soil organic matter (SOM) were determined by the potassium dichromate–sulfuric acid oxidation method followed by titration [36].

### 2.4. Soil Fauna Extraction, Identification and Counting

Soil fauna were collected using complementary sampling techniques, including hand sorting, monolith sampling and the Tullgren extraction method, following previously published protocols with minor modifications [37,38]. To reduce double counting, hand sorting was conducted first within a marked 1 m × 1 m area in each plot for 30 min, and all visible surface-active individuals were removed and preserved before soil monoliths were collected. The monolith sub-samples (25 cm × 25 cm to 15 cm depth, including litter) were then excavated from within the same marked area but from undisturbed positions. Monolith samples were placed under a 50 W electric light for 48 h in Tullgren extractors in the laboratory, causing soil fauna to move downward through the sieve and into beakers containing 70% ethanol (Figure S3, see Supplementary Materials). The soil fauna moved through the sieve and the funneled slopes due to the heat of the bulb and reached the beaker containing 70% ethanol. The soil fauna collected were counted and examined using a stereomicroscope (JSZ8, stereomicroscope, Nanjing Jiangnan Yongxin Optics Co., Ltd., Nanjing, China). Identification was carried out up to the order level using [39,40] and the online site iNaturalist.org (https://www.inaturalist.org). Abundance and distribution of each soil faunal group were expressed in individuals per m^2^.

### 2.5. Data Analysis

Order-level faunal abundance was computed with the following formula:

(1) N = n/A

where *N* is the abundance (individuals/m^2^), *n* is the total number of individuals collected per sample, and *A* is the sampled area (m^2^).

Menhinick’s diversity index (*D*) was calculated to assess order-level richness using the following equation:

(2) D=S/√N

where *S* is the total number of order-level taxa recorded in each plot and *N* is the total number of individuals.

Order-level diversity was computed using Shannon’s Diversity Index (*H*) [41].

(3) H=−Σpi ∗ ln(pi)

where *pi* represents the proportion of individuals belonging to the *i*th order relative to the total number of individuals in the plot.

Meanwhile, order-level evenness was calculated using Pielou’s Evenness Index (*EH*):

(4) EH=H/ln(S)

where *H* is the Shannon–Wiener diversity index and *S* is the total number of order-level taxa recorded in each plot.

### 2.6. Statistical Analysis

The obtained data were assessed for normality using the Shapiro–Wilk test with Lilliefors correction and for homogeneity of variances using Levene’s test. When the assumptions were met, differences among treatments were evaluated using analysis of variance (ANOVA) followed by Tukey’s HSD multiple comparison test. To account for the increased risk of Type I errors associated with multiple testing across numerous dependent variables, false discovery rate (FDR) correction using the Benjamini–Hochberg procedure was applied [42], and adjusted *p*-values were used to determine statistical significance (*p* < 0.05). Redundancy analysis (RDA) was applied to analyze the relationships between soil fauna community composition and measured soil physicochemical properties. All statistical analyses were carried out using SPSS, version 22.0. Figures were drawn using GraphPad Prism 10 software and Origin 2023b.

## 3. Results and Discussions

### 3.1. Effect of Different Mulch Treatments on Soil Physicochemical Properties

#### 3.1.1. Soil Temperature

**Figure 2:**
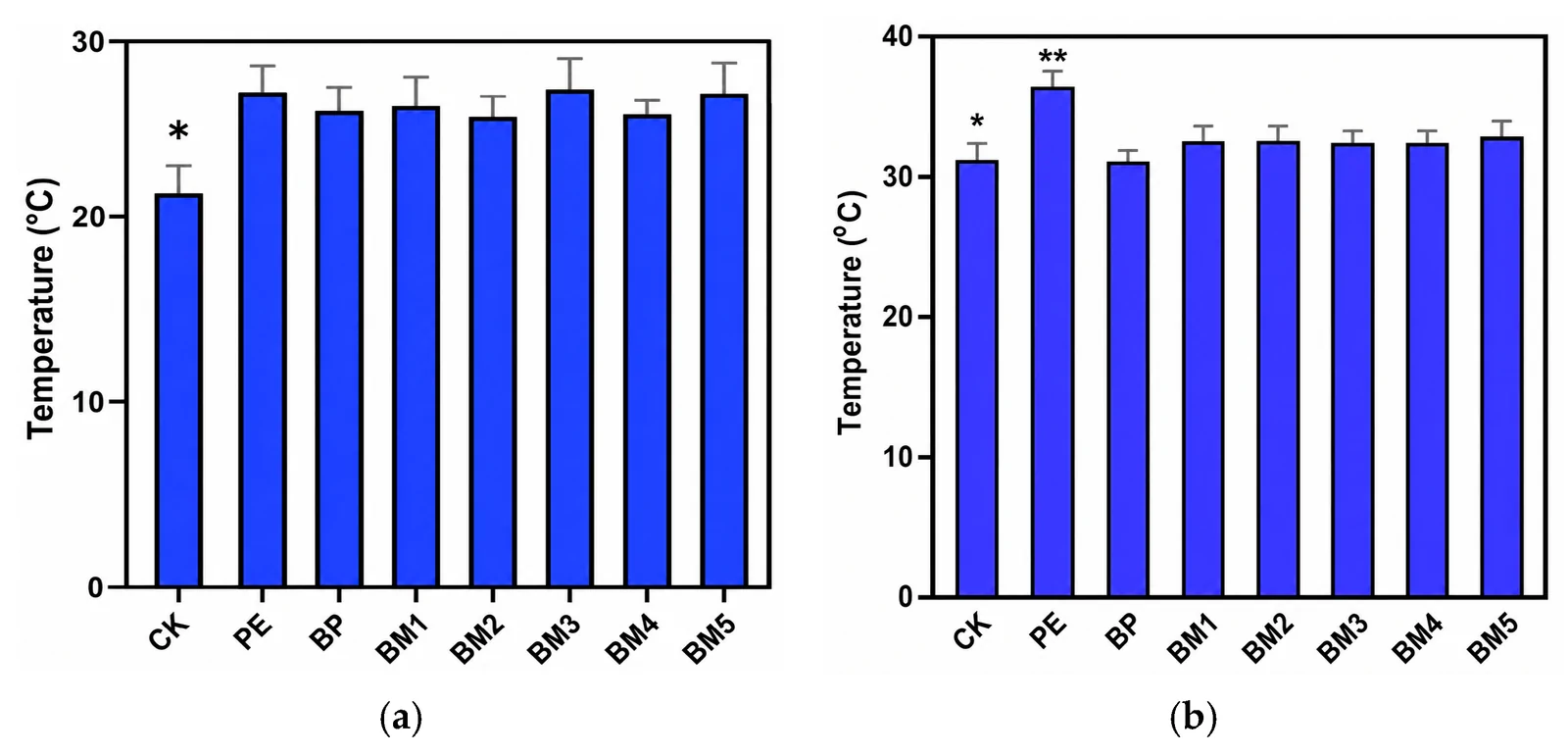
Soil temperature (°C) under different mulch treatments in comparison with control where asterisks “*” and “**” indicate significant differences at (*p* < 0.001) and (*p* < 0.01), respectively, during (**a**) growing stage and (**b**) harvesting stage.

In general, film mulching significantly increased (*p* < 0.001) soil temperature during both the growing and harvesting stages, compared to CK (Figure 2a), while BP (*p* < 0.001) and BMs (*p* < 0.01) significantly reduced soil temperature at the harvesting stage, compared to PE (Figure 2b).

Our result aligns with findings of [43], where soil temperature was similar in BM and PE, and temperatures were all higher than in CK before degradation of biodegradable films, while the soil temperatures after the degradation of the biodegradable films were similar in BM and CK, but both were lower than in PE. This indicated that the heating effect of biodegradable film mulching was reduced due to the fragmentation process at the harvesting stage, also confirmed by [44], while the BP treatment showed lower soil temperature compared to BMs because the BP was quickly degraded in the soil environment as compared to the BMs at the study site. This finding is supported by [45], who stated that biodegradable paper is much more degradable than biodegradable plastics.

#### 3.1.2. Soil pH and Electrical Conductivity

Under no mulch, the soil pH of CK was significantly higher (*p* < 0.05), compared with film mulching, during growing and harvesting stages (Table 1).

Our result is supported by [46], where plastic film mulching significantly lowered soil pH compared with no mulching. There were no significant differences observed in soil pH among different types of mulch treatments used except PE in the growing stage and BM1 in the harvesting stage. However, the soil pH was significantly lowered during the harvesting stage (*p* < 0.001) in almost all mulch treatments, compared to the growing stage. The lowered soil pH under film mulching at late stages was due to the comparatively high nitrate under plastic film mulching. In addition, soil electrical conductivity was significantly lowered in all mulch treatments compared to CK (*p* < 0.01), both in growing and harvesting stages. Studies have confirmed that film mulching can reduce water evaporation; as a result, the salinity of the mulched soil gets lowered, which may be the reason why electrical conductivity in the mulched soil is reduced compared to CK [47].

#### 3.1.3. Soil Water Content and Soil Organic Content

Water content was increased by all mulch treatments both during the growing and harvesting stages, compared to CK, while water content under BMs and BP was reduced during the harvesting stage, compared to PE (Table 1). The differences in water content in both stages were not statistically significant. Our findings indicate that moisture content in soil under treatments PE and BM was similar before degradation, but higher than CK, while after degradation it ranked as PE > BM > CK. The most possible cause was that film mulching significantly lowered soil evaporation and enhanced soil water conservation at early stages [48], while the lower water content under BMs and BP in late stages was due to the reduction in soil moisture conservation with the degradation of biofilm, as confirmed by [49,50]. Our study showed a decrease in water content under BP at both stages compared to BMs, because BP was unable to conserve water at the study site. This may be due to the quicker degradation of paper mulching compared to plastic mulching [45]. The soil organic carbon (SOC) and soil organic matter (SOM) were not significantly affected in all mulch treatments compared to CK, both in growing and harvesting stages, with the exception of the PE (*p* < 0.01). This demonstrates that film mulching had no impact on soil organic content in the short term [46]. The higher soil organic content in BMs and BP than in PE at the harvesting stage may be associated with biodegradable-material decomposition and altered microclimate, but direct carbon-contribution pathways require longer-term mass-balance or isotope-tracing experiments.

### 3.2. Effects of Different Mulch Treatments on Soil Fauna

A total of 5221 soil faunal individuals belonging to three phyla, four classes, and five orders were found in the study area (Table 2).

The prevalent orders were Stylommatophoran (33.19%) and Haplotaxidan (32.37%) followed by Coleopteran (17.89%), while Arachnid (7.45%) and Dipteran (6.05%) were least present in the rice field. The major soil fauna in the Stylommatophora order were snails, in the Haplotaxida order were earthworms, in the Coleoptera order were beetles, in the Arachnida order were spiders, and in the Diptera order were fly larvae and pupae, respectively (Figure S4, see Supplementary Materials). The overall soil faunal abundance during the six-month cropping system followed the order CK > BP > BM5 ≈ BM1 ≈ BM2 ≈ BM4 > BM3 > PE.

#### 3.2.1. Effects on Soil Fauna Abundance

**Figure 3:**
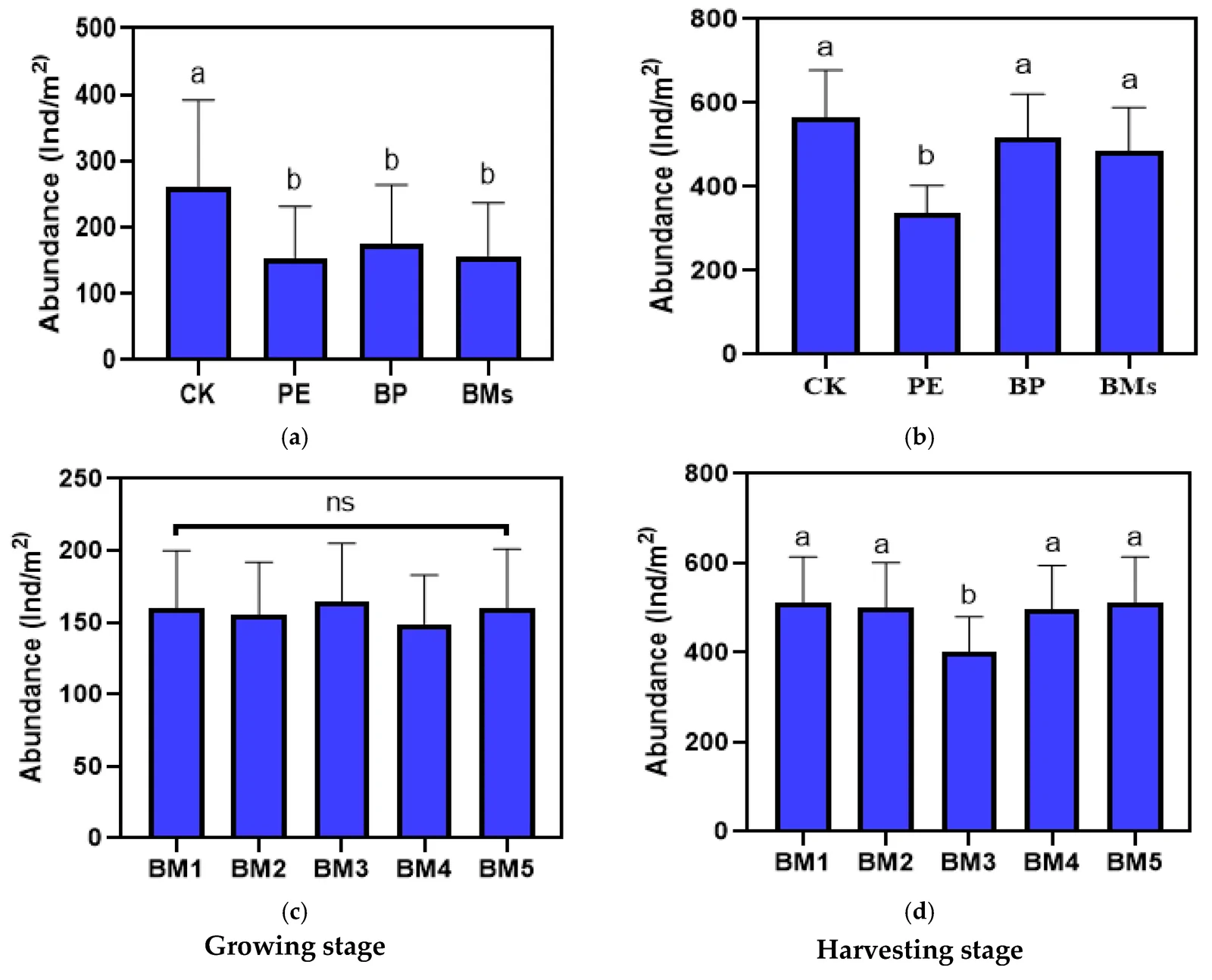
Abundance of soil fauna organisms (mean ± standard deviation) under different mulch treatments where different letters indicate significant differences at *p* < 0.05, and ns indicates no significant difference: (**a**,**c**) growing stage and (**b**,**d**) harvesting stage. The abundance of soil fauna is expressed as individuals per m^2^ (Ind/m^2^).

The total abundance of soil fauna under all of the mulch treatments was significantly reduced by 39% compared to CK (*p* < 0.05) during the growing stage (Figure 3a), but no significant differences were found between various kinds of BMs used (Figure 3c). All mulch treatments significantly reduced earthworm and snail abundance compared with CK (*p* < 0.05). Among the mulch treatments, BP maintained significantly higher earthworm abundance than PE and most BM treatments (Table S4, see Supplementary Materials). However, during the harvesting stage, BP and BMs significantly conserved soil fauna abundance compared with PE mulch (*p* < 0.05), showing increases of approximately 54% and 45%, respectively (Figure 3b). No significant differences were observed among the different BM treatments, except for BM3 (Figure 3d). Among the mulch treatments, BP (117.33 ± 4.44) and BM5 (112.00 ± 3.24) maintained relatively higher earthworm abundance compared with PE. (Table S5), while snail abundance significantly decreased under mulch treatments, particularly under PE (85.33 ± 2.24). In contrast, beetles and spiders responded positively to mulch treatments compared with CK, while dipteran abundance showed relatively minor variation, with BM4 supporting the highest abundance (53.33 ± 3.60) and BM5 the lowest (32.00 ± 2.00) (Table S5, see Supplementary Materials).

Plastic pollution is a serious global issue, raising its effects on soil fauna [51,52]. To the best of our knowledge, only limited studies have assessed the response of soil fauna to plastic pollution, especially to film mulching. In the present study, the lower faunal abundance observed under mulch treatments during the growing stage is interpreted primarily as a response to altered soil microclimatic conditions, particularly changes in temperature and moisture regimes. Soil fauna responses to warming are strongly regulated by soil moisture availability and taxon-specific ecological preferences. For example, earthworm activity may increase under moderate warming when adequate soil moisture is maintained, whereas elevated temperature combined with reduced moisture availability can negatively affect earthworm performance and decrease the abundance of moisture-sensitive invertebrates [26,27,28].

Temperature is a critical environmental factor influencing plant growth and soil animal survival [53,54]. In our study, soil temperature was significantly higher under all mulch treatments during the growing stage (Figure 2), which coincided with a significant reduction in soil faunal abundance compared with CK (Figure 3a). The decline in faunal abundance may reflect reduced habitat suitability and changes in faunal activity patterns under warmer soil conditions. During the harvesting stage, BP and BMs maintained relatively lower soil temperatures than PE mulch (Figure 2), which may have contributed to the preservation of soil faunal abundance under these treatments compared with PE mulch (Figure 3b). These results are consistent with previous studies showing that soil-dwelling organisms experienced greater stress under PE mulch than biodegradable mulches due to increased soil temperature and altered soil environments [55,56]. In addition to thermal stress, reduced soil organic content (SOC and SOM) under PE mulch (Table 1) may have further contributed to the decline in soil fauna abundance by limiting food resources and habitat quality. Previous studies have shown that soil organic matter depletion can substantially reduce earthworm abundance due to decreased resource availability [57]. Thus, the combined effects of elevated soil temperature and reduced organic matter availability may explain the lower faunal abundance under PE mulch, whereas BMs and BP provided more favorable conditions for sustaining soil fauna communities.

#### 3.2.2. Effects on Soil Faunal Community Indices

**Figure 4:**
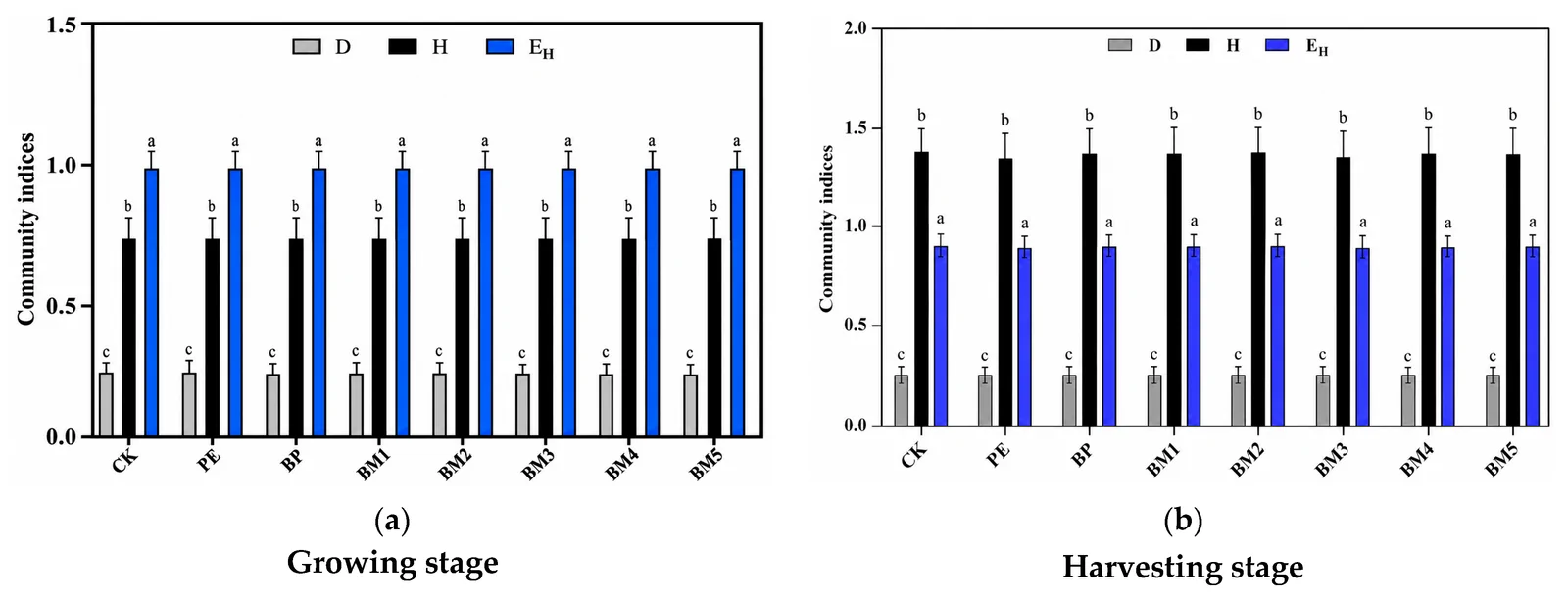
Richness (D), diversity (H), and evenness (E_H_) of the soil faunal community (mean ± standard deviation) under different film mulch treatments where different letters indicate significant differences at (*p* < 0.05) during (**a**) growing stage and (**b**) harvesting stage.

The community indices—richness index, diversity index, and evenness index—were calculated to analyze the comparable effects of different mulch treatments on soil faunal groups. Interestingly, there were no significant differences observed in richness index (*D*), diversity index (*H*) or evenness index (*E_H_*) of soil faunal groups between PE, BMs and BP during growing and harvesting stages (Figure 4). This indicated that short-term mulching had no impacts on soil faunal richness, diversity, or evenness. However, all treatments showed the lower diversity index and higher evenness index at the growing stage (Figure 4a). In contrast, all treatments showed a lower evenness index and higher diversity index at the harvesting stage (Figure 4b). This finding is in agreement with study of [58], where evenness index value at the growth stage of rice was fairly high and diversity index had the highest value at later stages.

#### 3.2.3. Effects on Soil Faunal Distribution

**Figure 5:**
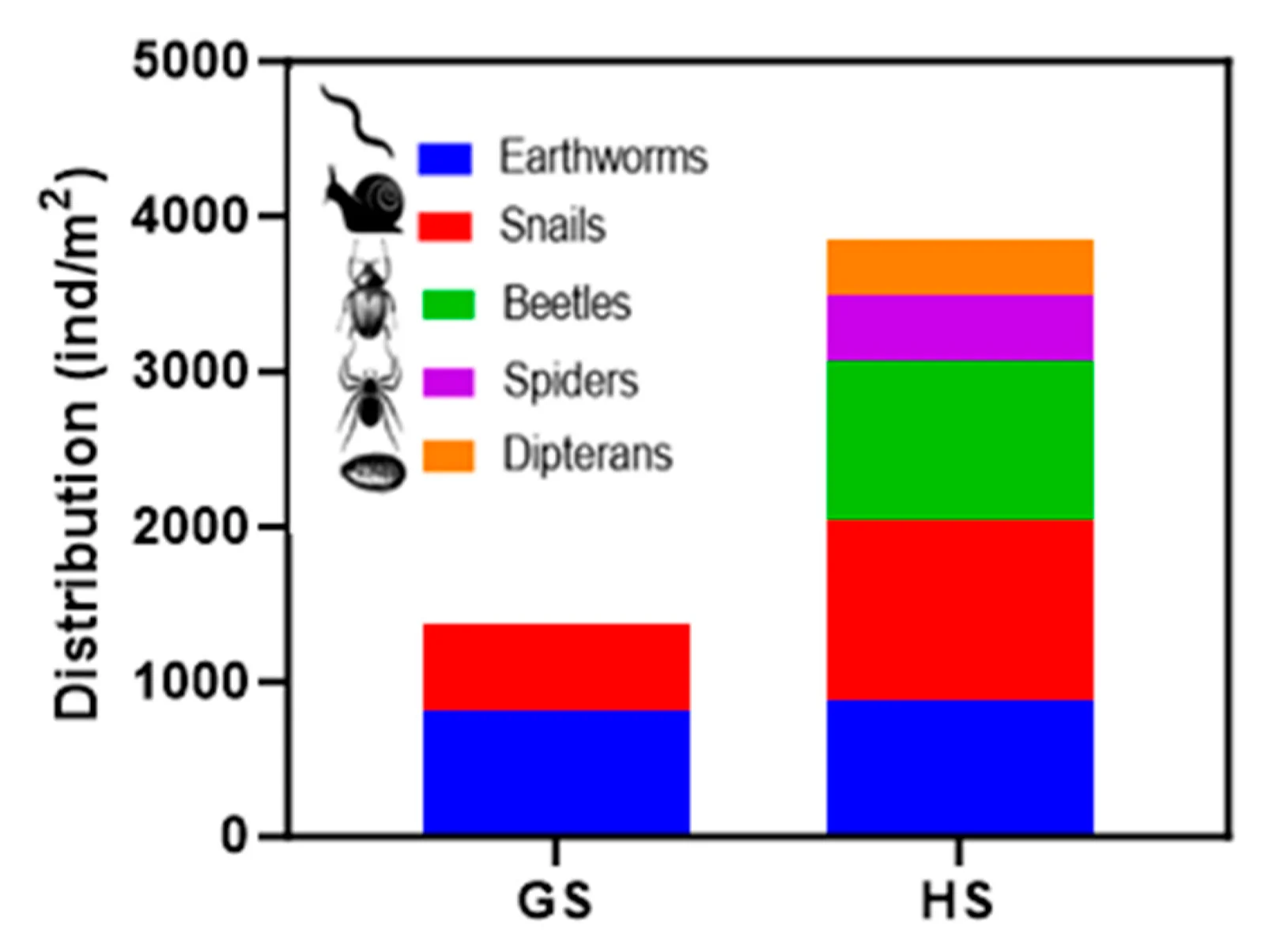
Soil fauna distribution at GS (growing stage) and HS (harvesting stage). The distribution of soil fauna is expressed as individuals per m^2^ (Ind/m^2^).

**Figure 6:**
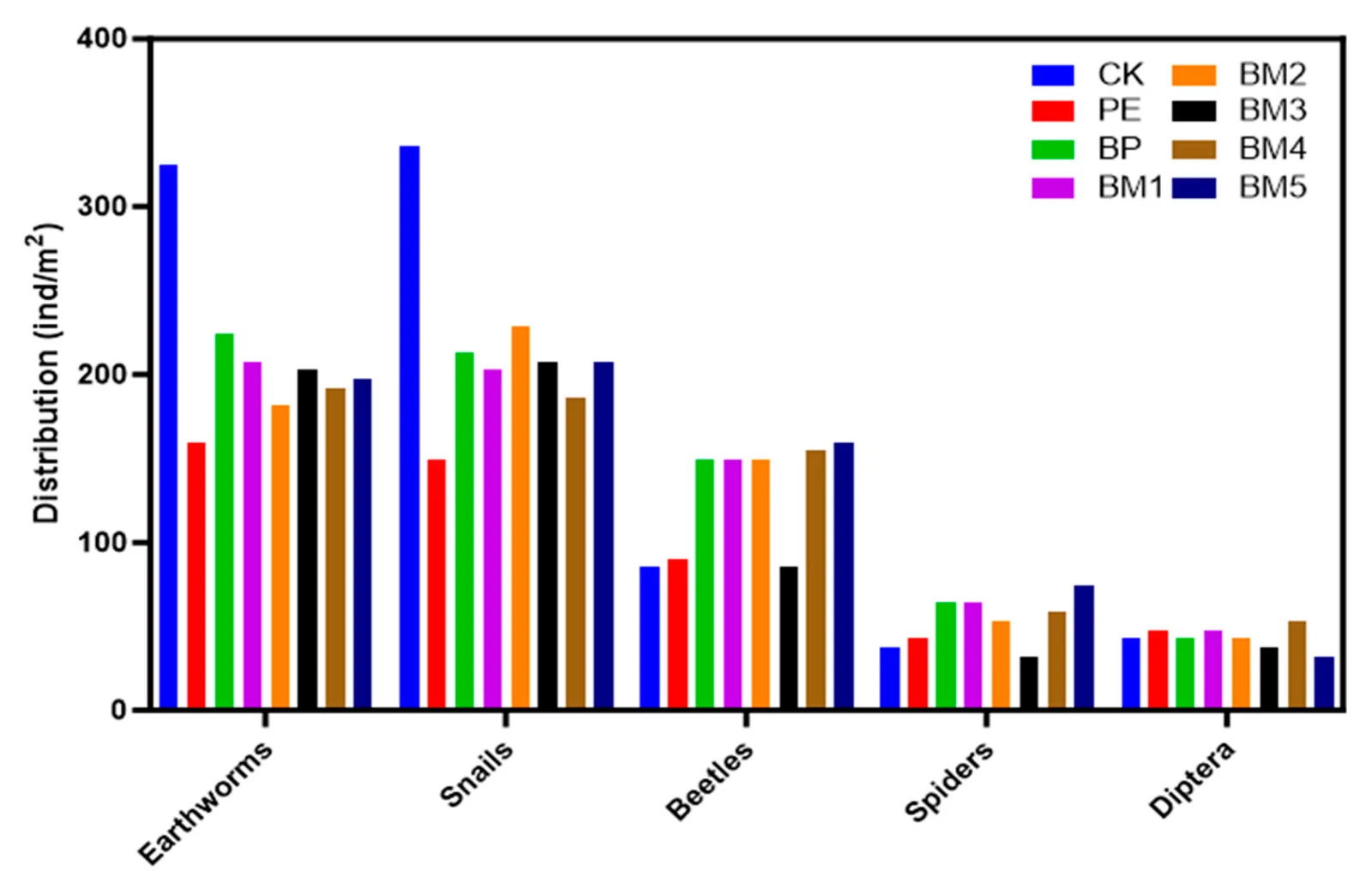
Distribution of major soil faunal groups under different mulch treatments. The distribution of soil fauna is expressed as individuals per m^2^ (Ind/m^2^).

The distribution of the major groups of soil fauna differed between the growing and harvesting stages (Figure 5). Earthworms and snails were more abundant species observed both in the growing and harvesting stages. However, beetles, spiders, and dipteran (larvae and pupae) were observed only during the harvesting stage, because the older the rice, the more insects are found in the field. This finding is supported by [58] who stated that fields provide surplus niches for insects at later stages, permitting insect populations to dominate at this stage. The presence of weeds during later stages also serves as a host for many insects in the field [59]. The other reason for their absence during the growing stage may be attributed to the high temperature stress, as these species are active and mobile, so they likely “hid or escaped” during the growing stage. The distribution of the major groups of soil fauna was also influenced by different mulch treatments (Figure 6) because of corresponding changes in the architecture and structure of root systems of rice. This is in agreement with an earlier study of Huang et al. [60], who found that film mulching can affect the soil food webs due to changes in the structure and functions of roots. Earthworms and snails were more impacted by mulch treatments compared to CK due to their low mobility and soft bodies, making them highly vulnerable to desiccation during high temperatures, as confirmed by [61]. In contrast, we observed fewer impacts of film mulching on insects compared to CK, as they possess a thick cuticle and are resistant to desiccation, as observed for beetles [62]. Radermacher et al. documented in their studies that insects rely on rice carbon as a source of food [63]. The greater abundance of beetles, spiders, and dipterans under BMs and BP in particular compared with PE may be associated with improved soil conditions, which can enhance habitat quality and resource availability for soil fauna. In our study, the higher SOC and SOM contents under BMs and BP may have contributed to more favorable soil habitats, thereby supporting greater faunal abundance. Previous studies have reported that film mulching can promote crop growth and increase carbon inputs through greater incorporation of crop residues into the soil, potentially enhancing soil faunal communities [64]. Although biodegradable mulches are composed of carbon-based polymers with relatively high carbon contents (~50%) [65], their direct contribution to soil carbon pools and utilization by soil fauna remain uncertain. Therefore, the higher abundance of soil fauna under BMs and BP is more likely related to their effects on soil environmental conditions, including temperature, moisture, and organic matter dynamics, rather than direct utilization of mulch-derived carbon [26,28]. In contrast, the higher soil temperatures observed under PE may have created unfavorable conditions for active and mobile insects, potentially reducing their activity and abundance compared with BMs and BP.

### 3.3. Multivariate Analysis

**Figure 7:**
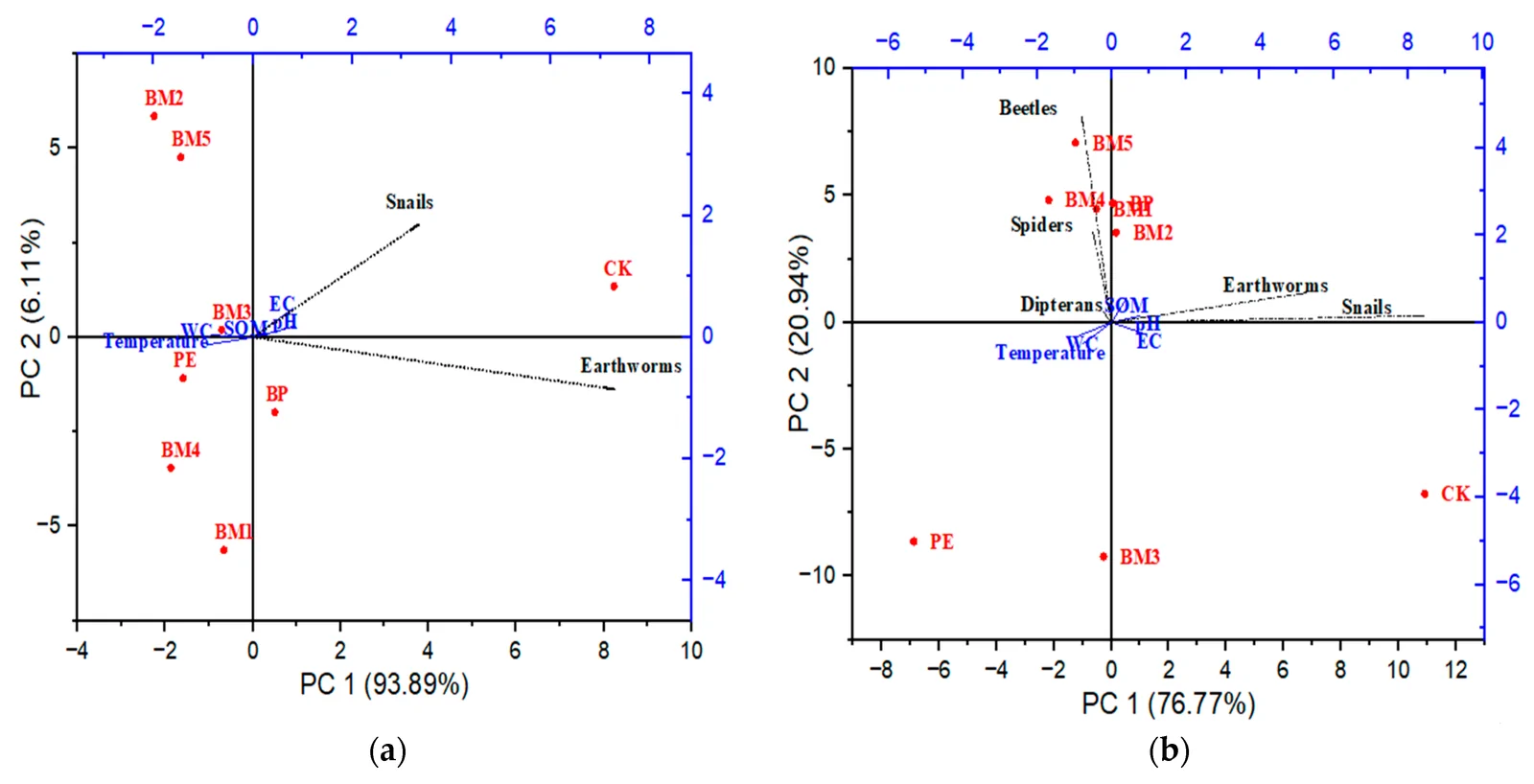
Redundancy analysis (RDA) of order-level soil-faunal groups in relation to measured soil variables during (**a**) growing stage and (**b**) harvesting stage. Blue arrows represent soil physicochemical parameters labeled as water content (WC), soil organic matter (SOM), soil electrical conductivity (EC), soil pH and soil temperature. Red dots represent different mulch treatments labeled as polyethylene (PE), biodegradable paper (BP), biodegradable mulches (BMs1-5) and no-mulch control (CK). Black arrows represent soil faunal groups.

Redundancy analysis (RDA) was used to explore associations between the measured soil variables and soil fauna abundance. During the growing stage, the first two axes explained 93.89% and 6.11% of the displayed variation, respectively (Figure 7a). During the harvesting stage, the first two axes explained 76.77% and 20.94%, respectively (Figure 7b).

The RDA revealed a positive association between soil fauna and the CK treatment during the growing stage (Figure 7a), suggesting that differences in soil faunal abundance were associated with variations in the measured soil environmental parameters under the different mulch treatments. Among these parameters, soil temperature, water content and soil organic matter showed strong associations with faunal distribution patterns. Previous studies have reported that high soil temperatures may create less favorable conditions for many soil Cfaunas [66]. Therefore, the weaker association of soil fauna with the mulched treatments observed may be related to the relatively higher soil temperatures under these treatments. At the harvesting stage, no clear discrimination was observed among CK, BP, and the BM treatments (except BM3) as compared to PE (Figure 7b). This lack of discrimination was associated with more favorable environmental conditions under BMs and BP, including decreased soil temperature and increased soil organic matter (SOM).

## 4. Conclusions

This study investigated the short-term effects of conventional polyethylene (PE), biodegradable film mulches (BMs), biodegradable paper (BP) and no mulch (CK) on specific soil physicochemical properties and soil fauna communities in a six-month cropping system. All mulch treatments altered soil physicochemical conditions and reduced soil faunal abundance during the growing stage, with an average decline of 39% compared with CK. However, during the harvesting stage, soil faunal abundance under BP and BMs was higher than under PE, suggesting a lower disturbance potential of biodegradable mulches under later crop stages. Soil hydrothermal condition and soil organic matter were identified as key factors associated with variations in soil faunal abundance. Overall, biodegradable mulches, particularly paper-based mulch, showed potential for maintaining soil faunal abundance compared with PE under short-term field conditions. However, these conclusions are limited to a single cropping cycle and the measured parameters. Long-term and multi-site studies across diverse croplands and soil types are needed to further evaluate the effects of biodegradable and conventional mulching practices on soil biodiversity and ecosystem functioning.

## Figures and Tables

**Table 1:** Soil physicochemical properties under different mulch treatments in rice field, Biasha County.

GS	CK	PE	BP	BM1		BM2	BM3	BM4	BM5
pH	7.18 ± 0.02 ^a^	6.42 ± 0.08 ^ab^	6.74 ± 0.03 ^b^	6.63 ± 0.05 ^b^		6.57 ± 0.03 ^b^	6.77 ± 0.05 ^b^	6.74 ± 0.05 ^b^	6.82 ± 0.03 ^b^
EC (mS/cm)	25.96 ± 3.03 ^a^	12.09 ± 0.35 ^ab^	16.51 ± 0.51 ^b^	17.05 ± 1.02 ^b^		18.64 ± 0.50 ^b^	17.58 ± 1.33 ^b^	16.65 ± 1.73 ^b^	19.88 ± 0.72 ^b^
WC (%)	1.35 ± 0.34 ^a^	2.18 ± 1.20 ^a^	1.57 ± 0.74 ^a^	2.27 ± 1.00 ^a^		2.06 ± 1.22 ^a^	2.15 ± 1.46 ^a^	2.23 ± 2.37 ^a^	2.51 ± 1.08 ^a^
SOC (g/kg)	9.29 ± 1.94 ^a^	9.49 ± 2.26 ^a^	8.73 ± 2.07 ^a^	7.67 ± 2.32 ^a^		8.45 ± 2.01 ^a^	8.33 ± 2.14 ^a^	8.16 ± 1.26 ^a^	10.04 ± 2.05 ^a^
SOM (g/kg)	16.01 ± 3.34 ^a^	16.36 ± 3.90 ^a^	15.05 ± 3.57 ^a^	13.22 ± 4.00 ^a^	14.57 ± 3.47 ^a^	14.36 ± 3.69 ^a^	14.07 ± 2.17 ^a^	17.31 ± 3.53 ^a^	
HS	CK	PE	BP	BM1		BM2	BM3	BM4	BM5
pH	6.92 ± 0.09 ^b^	6.40 ± 0.02 ^a^	6.68 ± 0.05 ^a^	6.06 ± 0.22 ^ab^		6.46 ± 0.25 ^a^	6.30 ± 0.19 ^a^	6.15 ± 0.13 ^a^	6.55 ± 0.10 ^a^
EC (mS/cm)	18.69 ± 0.89 ^b^	9.37 ± 2.33 ^a^	8.25 ± 1.28 ^a^	12.92 ± 2.38 ^a^		10.75 ± 0.72 ^a^	11.90 ± 1.77 ^a^	11.63 ± 2.44 ^a^	12.91 ± 1.97 ^a^
WC (%)	1.25 ± 0.87 ^a^	2.84 ± 2.49 ^a^	1.71 ± 0.64 ^a^	1.88 ± 0.54 ^a^		1.86 ± 0.52 ^a^	1.86 ± 0.29 ^a^	1.83 ± 0.52 ^a^	1.92 ± 0.59 ^a^
SOC (g/kg)	9.00 ± 1.11 ^a^	6.58 ± 1.08 ^b^	9.67 ± 1.22 ^a^	9.62 ± 1.17 ^a^		9.58 ± 1.30 ^a^	9.85 ± 1.22 ^a^	9.09 ± 1.25 ^a^	10.09 ± 1.17 ^a^
SOM (g/kg)	15.52 ± 1.91 ^a^	11.34 ± 1.86 ^b^	16.67 ± 2.10 ^a^	16.58 ± 2.02 ^a^	16.51 ± 2.24 ^a^	16.98 ± 2.10 ^a^	15.66 ± 2.16 ^a^	17.39 ± 2.02 ^a^	

Note: Values followed by different small letters within rows indicate significant difference at *p* < 0.05. GS: growing stage; HS: harvesting stage; CK: control; PE: polyethylene; BP: biodegradable paper; BMs1–5: biodegradable mulches.

**Table 2:** Soil fauna distribution and abundance (individuals/m^2^) under different mulch treatments.

Common Names	Groups			Distribution Across Treatments								Total Abundance
	Phylum	Class	Order	CK	PE	BP	BM1	BM2	BM3	BM4	BM5	
Earthworms	Annelida	Clitellata	Haplotaxida	325	160	224	208	181	203	192	197	1691
Snails	Mollusca	Gastropoda	Stylommataphora	336	149	213	203	229	208	187	208	1733
Beetles	Arthropoda	Insecta	Coleoptera	85	91	149	149	149	85	155	160	1024
Spiders	Arthropoda	Arachnida	Arachnid	37	43	64	64	53	32	59	75	427
Dipterans	Arthropoda	Insecta	Diptera	43	48	43	48	43	37	53	32	347
(larva and pupae)		Overall distribution		826	491	693	672	655	565	646	672	5221

CK: control; PE: polyethylene; BP: biodegradable paper; BMs1–5: biodegradable mulches.

## Data Availability

The original contributions presented in this study are included in the article/Supplementary Materials. Further inquiries can be directed to the corresponding authors.

## Supplementary Materials

The following supporting information can be downloaded at: https://www.mdpi.com/article/10.3390/toxics14080651/s1. Table S1: Basic physical and chemical properties of soil in the field-test site; Tabe S2: Soil temperature (°C) and relative humidity (% RH) at study site; Table S3: Characteristics of the film mulches used at the study site; Table S4: Soil faunal abundance (mean ± SD) under different mulch treatments at harvesting stage; Table S5: Soil faunal abundance (mean ± SD) under different mulch treatments at harvesting stage; Figure S1: The site photos of cropping field covered with film mulching (A) study field; (B, C) polyethylene and biodegradable mulches; (D) biodegradable paper; (E) No-mulch, control. The person appearing in Figure S1B is corresponding author of this manuscript. Therefore, no copyright or per-mission issues are associated with this image; Figure S2: Schematic layout of the field experiment; Figure S3. Soil fauna extraction method by using Tullgren extractors; Figure S4: Discovered soil fauna during present study (A) Beetle (B) Spi-der (C) Snail (D) Earth-worm (E, F) Larvae and Pupae of Flies.
