# Supplementary material for: Evaluating the Effects of Conventional Polyethylene and Biodegradable Film Mulching on Soil Fauna Abundance and Diversity in a Rice Cropping System
Source: Toxics. 2026 Jul 24;14(8):651. doi: 10.3390/toxics14080651 (PMC13517243; doi:10.3390/toxics14080651)
Supplement: Supplementary file 1 [file toxics-14-00651-s001.zip › toxics-4393113-supplementary.pdf]

Supplementary materials

Table S1. Basic physical and chemical properties of soil in the field-test site

BD ($g.cm^{-3}$)	Soil pH	SOC ($g·kg^{-1}$)	SOM ($g·kg^{-1}$)	TN ($g·kg^{-1}$)	AN ($mg·kg^{-1}$)	AP ($mg·kg^{-1}$)
1.29±0.09	7.18±0.02	9.29±1.94	16.01 ± 3.34	1.41±0.02	52.35±1.01	6.67±0.62

Abbreviations: BD, bulk density; SOC, soil organic carbon; SOM, soil organic matter; TN, total nitrogen; AN, available nitrogen; AP, available phosphorous

Tabe S2. Soil temperature（°C) and relative humidity（% RH) at study site

Treatment	Parameter	February	March	April	May	June
CK	Soil temperature（°C）	21.74	21.69	25.47	25.13	25.53
	Relative humidity（% RH）	40.89	40.08	39.85	37.45	37.05
PE	Soil temperature（°C）	22.00	22.69	26.20	27.77	30.97
	Relative humidity（% RH）	54.02	50.74	49.52	46.89	48.89
BP	Soil temperature（°C）	19.80	21.72	26.08	27.16	26.01
	Relative humidity（% RH）	50.89	50.45	45.03	43.86	40.16
BM1	Soil temperature（°C）	21.78	21.56	25.17	26.42	27.42
	Relative humidity（% RH）	46.75	49.11	48.70	47.66	46.66
BM2	Soil temperature（°C）	21.73	21.97	24.68	25.46	27.46
	Relative humidity（% RH）	49.79	56.83	54.82	41.78	43.78
BM3	Soil temperature（°C）	23.09	24.08	25.96	26.36	27.86
	Relative humidity（% RH）	46.09	46.75	44.16	40.34	43.94

BM4	Soil temperature (℃)	20.90	22.30	24.90	26.07	27.97
	Relative humidity（% RH）	48.05	49.43	51.61	45.22	44.22

Note: Data for the BM5 treatment were unavailable due to equipment failure

Table S3. Characteristics of the film mulches used at the study site.

Mulch type with abbreviations	Composition	Company name	Thickness (μm)	Length (m)	Width (m)	Color
Non-biodegradable plastic (PE)	Polyethylene	SINOPEC (Beijing) Research Institute of Chemical Industry Co., Ltd. China	10	50	2	Black
Biodegradable plastic (BM1)	Polyglycolic acid	SINOPEC (Beijing) Research Institute of Chemical Industry Co., Ltd. China	10	50	2	Black
Biodegradable plastic (BM2)	Polylactic acid	SINOPEC (Beijing) Research Institute of Chemical Industry Co., Ltd. China	10	50	2	Black
Biodegradable plastic (BM3)	Poly(butylene adipate-co-terephthalate)	Shandong Qingtian Plastic Co., Ltd. China	10	50	2	Black
Biodegradable plastic (BM4)	Polyglycolic acid / Poly(butylene adipate-co-terephthalate)	Shanghai Hongrui Chemical Co., Ltd, China	10	50	2	Black
Biodegradable plastic (BM5)	Polylactic acid / Poly(butylene adipate-co-terephthalate)	Baishan Xifeng Plastics Co., Ltd. China	10	50	2	Black
Biodegradable paper (BP)	Kraft paper	Local shop in Haikou, Hainan, China	100	50	2	Brown

PE: Polyethylene, BMs1-5: Biodegradable mulches, BP: Biodegradable paper.

Table S4. Soil faunal abundance (mean ± SD) under different mulch treatments at harvesting stage.

Treatments	CK	PE	BP	BM1	BM2	BM3	BM4	BM5
Earthworms	160.00 ± 6.45[a]	90.67 ± 3.31[cd]	106.67 ± 3.88[b]	101.33 ± 3.08[bc]	80.00 ± 3.02[d]	96.00 ± 3.33[c]	90.67 ± 3.22[cd]	85.33 ± 4.95[d]
Snails	101.33 ± 5.33[a]	64.00 ± 3.31[c]	69.33 ± 3.81[bc]	58.67 ± 3.08[c]	74.67 ± 3.44[b]	69.33 ± 3.81[bc]	58.67 ± 3.08[c]	74.67 ± 3.44[b]

Note: Different superscript letters represent significant differences among treatments ($P < 0.05$), while treatments sharing the same letters are statistically similar.

Table S5. Soil faunal abundance (mean ± SD) under different mulch treatments at harvesting stage.

Treatments	CK	PE	BP	BM1	BM2	BM3	BM4	BM5
Earthworms	165.33 ± 6.88[a]	69.33 ± 2.24[c]	117.33 ± 4.44[b]	106.67 ± 3.08[bc]	101.33 ± 2.88[c]	106.67 ± 3.08[bc]	101.33 ± 2.88[c]	112.00 ± 3.24[bc]
Snails	234.67 ± 7.31[a]	85.33 ± 2.24[c]	144.00 ± 3.00[bc]	144.00 ± 3.00[bc]	154.67 ± 3.44[b]	138.67 ± 3.24[c]	128.00 ± 3.11[c]	133.33 ± 3.23[c]
Beetles	85.33 ± 4.24[c]	90.67 ± 2.44[c]	149.33 ± 3.44[b]	149.33 ± 3.44[b]	149.33 ± 3.44[b]	85.33 ± 2.11[c]	154.67 ± 3.80[ab]	160.00 ± 3.90[a]
Spiders	37.33 ± 2.24[cd]	42.67 ± 2.40[cd]	64.00 ± 2.90[ab]	64.00 ± 2.90[ab]	53.33 ± 2.22[bc]	32.00 ± 2.00[d]	58.67 ± 2.14[bc]	74.67 ± 3.00[a]
Dipterans	42.67 ± 3.24[bc]	48.00 ± 3.00[b]	42.67 ± 3.24[bc]	48.00 ± 3.00[b]	42.67 ± 3.24[bc]	37.33 ± 3.00[c]	53.33 ± 3.60[a]	32.00 ± 2.00[c]

Note: Different superscript letters represent significant differences among treatments ($P < 0.05$), while treatments sharing the same letters are statistically similar.

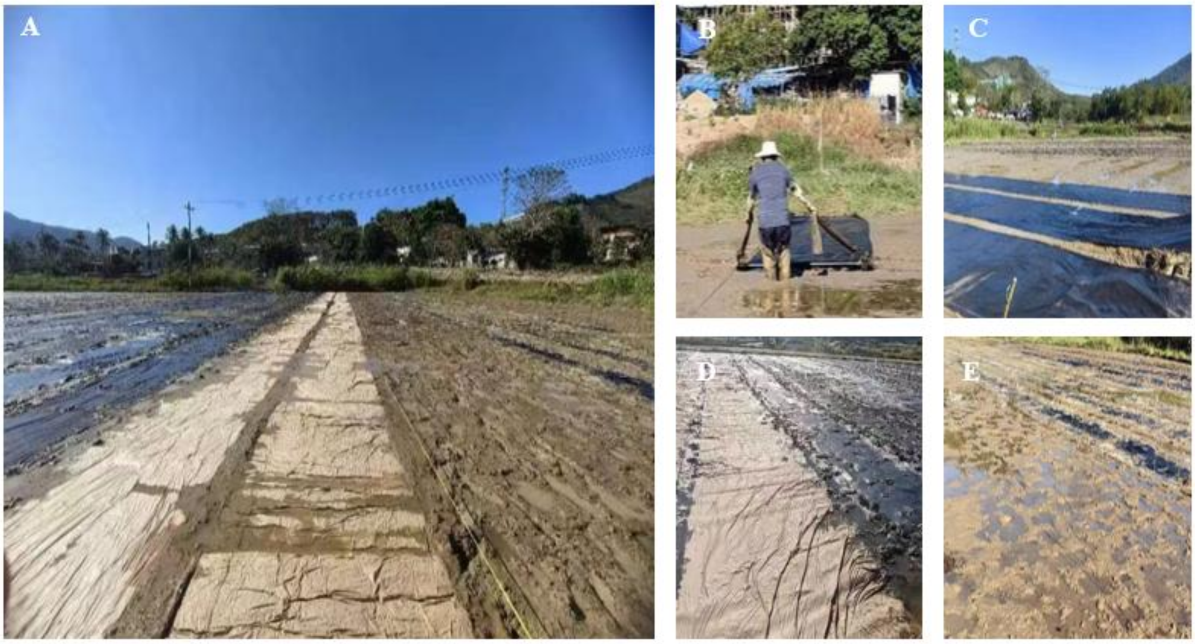

Figure S1. The site photos of cropping field covered with film mulching (A) study field; (B, C) polyethylene and biodegradable mulches; (D) biodegradable paper; (E) No-mulch, control. *The person appearing in Figure S1B is corresponding author of this manuscript. Therefore, no copyright or permission issues are associated with this image.*

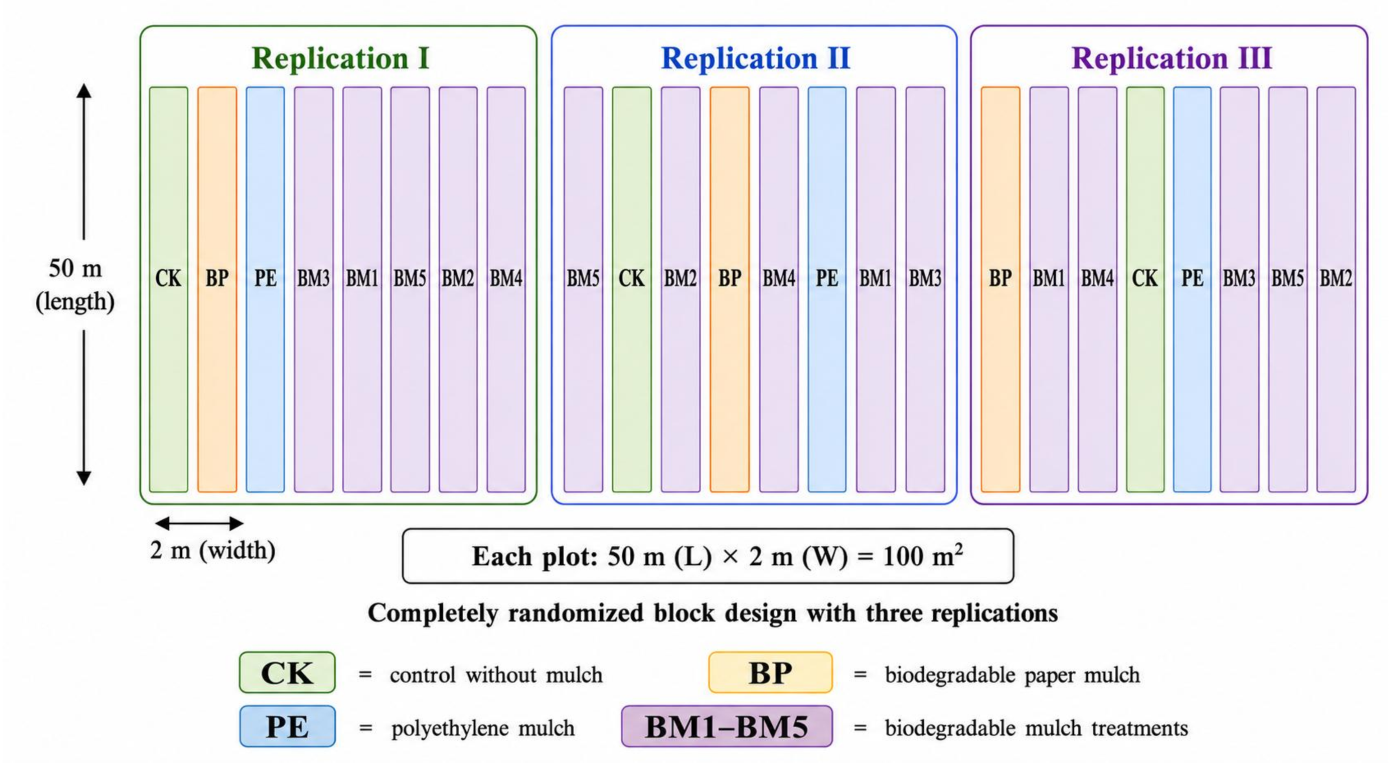

Figure S2. Schematic layout of the field experiment.

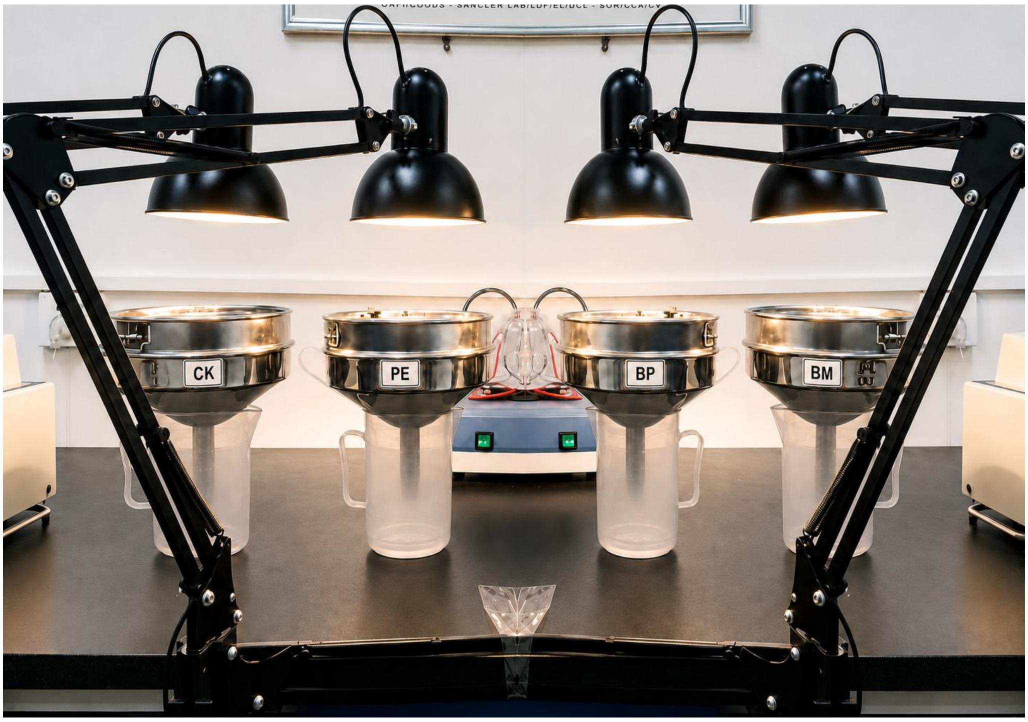

Figure S3. Soil fauna extraction method by using Tullgren extractors.

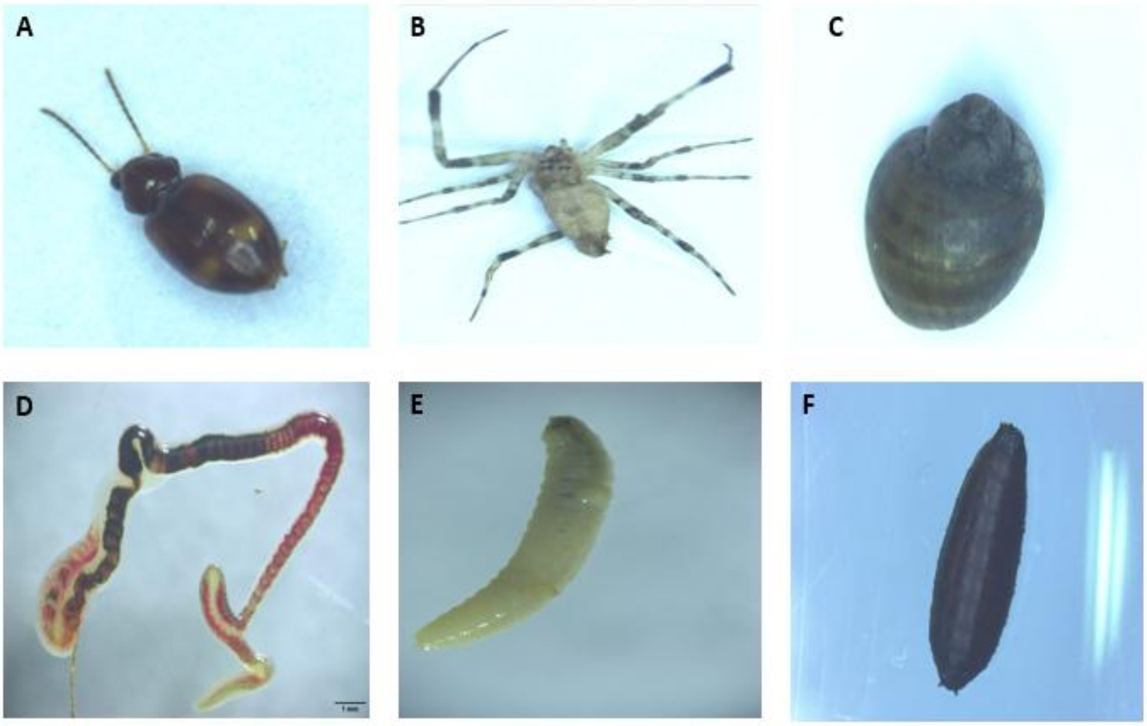

Figure S4. Discovered soil fauna during present study (A) Beetle (B) Spider (C) Snail (D) Earthworm (E, F) Larvae and Pupae of Flies.

https://doi.org/10.3390/xxxxx
